# High arsenic in rice is associated with elevated genotoxic effects in humans

**DOI:** 10.1038/srep02195

**Published:** 2013-07-22

**Authors:** Mayukh Banerjee, Nilanjana Banerjee, Pritha Bhattacharjee, Debapriya Mondal, Paul R. Lythgoe, Mario Martínez, Jianxin Pan, David A. Polya, Ashok K. Giri

**Affiliations:** 1 Molecular and Human Genetics Division, CSIR-Indian Institute of Chemical Biology, 4, Raja S. C. Mullick Road, Kolkata – 700 032, India; 2 School of Earth, Atmospheric and Environmental Sciences and Williamson Research Centre for Molecular Environmental Science, University of Manchester, M13 9PL, Manchester, UK; 3 Probability and Statistics Group, School of Mathematics, University of Manchester, M13 9PL, Manchester, UK; 4 Current address: Department of Physiology, Medical Science Building, Room 7-14B, University of Alberta, Edmonton, AB, T6G 2H7, Canada; 5 Current address: School of Environment & Life Sciences, University of Salford, Salford, UK

## Abstract

Arsenic in drinking water may cause major deleterious health impacts including death. Although arsenic in rice has recently been demonstrated to be a potential exposure route for humans, there has been to date no direct evidence for the impact of such exposure on human health. Here we show for the first time, through a cohort study in West Bengal, India, involving over 400 human subjects not otherwise significantly exposed to arsenic through drinking water, elevated genotoxic effects, as measured by micronuclei (MN) in urothelial cells, associated with the staple consumption of cooked rice with >200 μg/kg arsenic. Further work is required to determine the applicability to populations with different dietary and genetic characteristics, but with over 3 billion people in the world consuming rice as a staple food and several percent of this rice containing such elevated arsenic concentrations, this study raises considerable concerns over the threat to human health.

Chronic arsenic toxicity from ingestion of contaminated drinking water has been reported from many countries of the world and is an environmental problem of colossal proportions^1^ with a wide range of deleterious health impacts, including hyperpigmentation, keratosis, skin and internal cancers, and vascular diseases^1^,^2^,^3^,^4^. More than 3,000,000,000 people across the world consume rice as a staple food^5^. Arsenic contents of such rice varies widely, with most reported concentrations found in the range 20–900 μg/kg^5^. Recently rice has also been identified as a major exposure route^5^,^6^,^7^,^8^,^9^,^10^,^11^, as evidenced by observations of a strong association between rice consumption and urinary arsenic^12^,^13^. Indeed it is often the most important human exposure route where drinking water arsenic concentrations are less than 50 μg/L^8^,^9^,^10^. The relatively high proportion of the more toxic inorganic arsenic forms in rice^11^ together with high arsenic bioavailabilities^14^ and bioaccessibilities^15^ add to the increasing plausible concern that arsenic in rice could be a health threat to millions of people. Notwithstanding this, to our knowledge, there are to date no studies that demonstrate such deleterious health impacts in humans consuming high arsenic rice in the absence of exposure through drinking water. We therefore designed a study to determine if cooked rice arsenic content on its own is sufficient to give rise to genotoxic effects in humans.

## Results

### Demographic characteristics of the study participants

The summary demographic characteristics and exposure status of the entire study population based in rural West Bengal, India, are shown in Table 1. All the study groups (A–F), selected on the basis of equally spaced classification boundaries with respect to cooked rice arsenic, are similar with respect to their age and gender distribution, body weight, and also their tobacco usage. For all groups, the mean arsenic content of drinking water was between 3 and 6 μg/L and the mean drinking water intake between 2.9 and 3.8 L/day. Combined with mean cooked rice intakes of between 540 and 600 g/day, this means that arsenic exposure from drinking water contributed no more than 20% of total dietary exposure for any group, and less than 12% of total dietary exposure for the study population taken as a whole.

### Urinary arsenic and exposure from cooked rice

**Figure 1:**
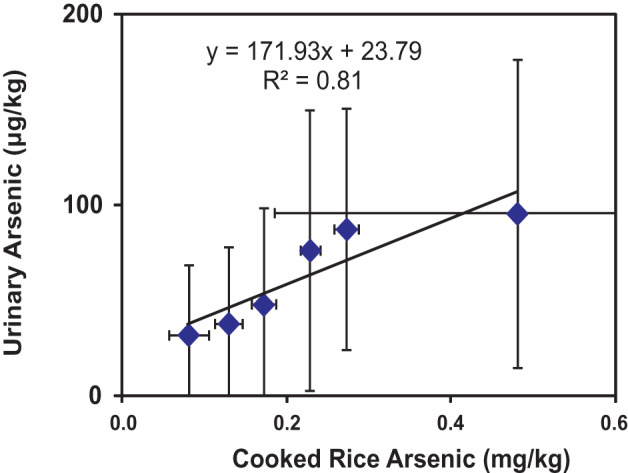
Cross-plot of mean urinary arsenic vs mean cooked rice arsenic. The linear best-fit trendline is indicative only. Error bars represent ± 1 standard deviation for each parameter for each exposure group (A–F).

There is a strong correlation (r^2^ = 0.81) between grouped urinary arsenic and cooked rice arsenic data (Table 1, Fig. 1) confirming the overwhelming importance of rice as the major dietary exposure route in the study population. This relationship was also found to varying degrees of fits to all of: males, females, tobacco-users, non-tobacco users, and participants from each of the 3 study areas.

### Genetic damage status as measured by micronucleus assay

**Figure 2:**
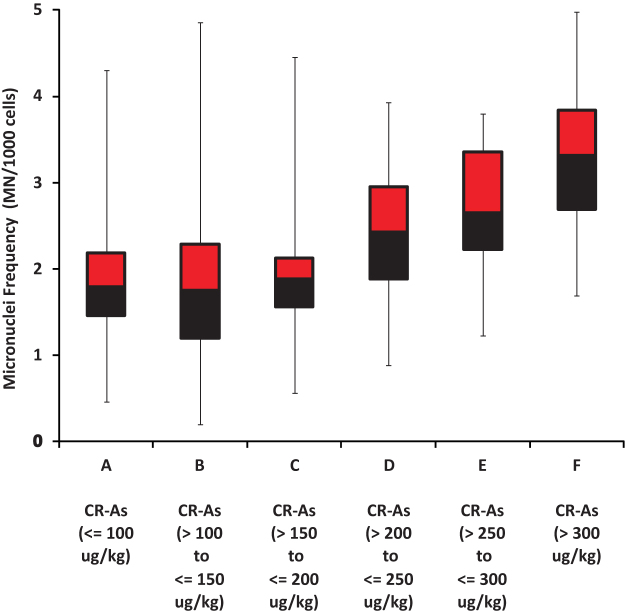
Urothelial cell genetic damage, as measured by frequency (per 1000 cells) of induction of micronuclei (MN), as a function of total arsenic concentration (CR-As) in consumed cooked rice, grouped as indicated. For this rural West Bengal population consuming rice as a staple, high arsenic in cooked rice is associated with elevated genotoxic effects. All groups with a mean As in cooked rice >200 μg/kg (D, E, F) have mean micronuclei frequencies (MN/1000 cells) significantly higher (p < 0.05) than those of the lower exposure groups (A, B, C). Numbers in each group as in Table 1.

For the whole cohort, MN ranged from 0.50 to 4.98, with a median of 1.91 and an inter-quartile range from 1.56 to 2.56. The whole cohort mean MN was 2.12 ± 0.89 (SD, n = 417). On a grouped basis, MN increased monotonically with mean cooked rice arsenic content from 1.85 ± 0.63 (SD, n = 113) for the lowest cooked rice arsenic group (A) up to 3.23 ± 0.93 (SD, n = 37) for the highest cooked rice arsenic group (F). Preliminary linear regression analysis, using the cooked rice arsenic groups as categorical variables and with adjustment for gender, body weight, tobacco usage and drinking water arsenic concentration, showed that groups with mean cooked rice arsenic >200 μg/kg (D, E & F) each showed significantly higher (p < 0.001) micronuclei frequencies than the lowest exposure group (A), with the coefficients for the predicted increased in MN for each group relative to the reference group (A) as follows: Group B: 0.05 (95% CI −0.15 to 0.25; p = 0.631; Group C: 0.11 (95% CI −0.11 to 0.33; p = 0.338); Group D: 0.66 (95% CI 0.35 to 0.96; p < 0.001); Group E: 0.83 (95% CI 0.50 to 1.15; p < 0.001) and Group F: 1.38 (95% CI 1.09 to 1.68; p < 0.001). Further preliminary statistical analysis (One-way ANOVA with Tukey-Kramer Multiple Pairwise Comparisons Test modified to account for unequal group sizes and variance) shows that groups with mean cooked rice arsenic >200 μg/kg (D, E & F) each have significantly higher (p < 0.05) induction of genetic damage compared to each of the groups with mean cooked rice arsenic < = 200 μg/kg (A, B & C), although a relatively low p(>~0.005) was required by the w/s test invoked in order to fully comply with the requirement of this test for within group normal distributions of MN. A more robust non-parametric analysis (Kruskal-Wallis test followed by Wilcoxon Rank Sum Test with continuity correction) confirmed the result that all the groups with mean cooked rice arsenic >200 μg/kg (D, E & F) showed significantly higher (p < 0.05) micronuclei frequencies than the lower exposure groups (A, B & C) (Kruskal-Wallis Rank Sum test (χ^2^ = 83.9113; df = 5; p < 2.2e-16); Wilcoxon Rank Sum tests for: groups A & D: W = 951; p = 3.654e-07; groups A & E: W = 695.5; p = 7.293e-07; groups A&F: W = 512.5; p = 6.077e-12; groups B & D: W = 1127; p = 4.651e-05; groups B & E: W = 862; p = 1.499e-05; groups B & F: W = 664.5; p = 1.864e-10; groups C & D: W = 840.5; p = 0.0002438; groups C & E: W = 586; p = 1.462e-05; groups C & F: W = 500.5; p = 3.138e-09) (Fig. 2).

No significant differences (p < 0.05) were found by either the modified Tukey-Kramer test or the more robust non-parametric Wilcoxon Rank Sum test between the group mean MN for any pairs of groups with cooked rice arsenic < = 200 μg/kg (A, B & C), but because of the small sample size, we are unable to determine if this reflects that there is no relationship between MN and cooked rice arsenic at these lower cooked rice arsenic concentrations or merely that our study had insufficient power to detect such a relationship. Similarly, the relatively small number of samples (n = 5) for which cooked rice arsenic exceeded 600 μg/kg means that we are unable to determine whether or not the relationship between cooked rice arsenic and MN for our study was more linear or sub-linear for these very high concentrations.

Notwithstanding these limitations, the highest rice arsenic content which has not been observed to be unequivocally associated with significantly increased genetic damage (Group C, Fig. 1) is 200 μg/kg, equivalent to 112 μg of arsenic solely from rice sources each day and, given the mean body weight of the study participants of 50.8 kg (Table 1), equivalent to a dosage of 2.2 μg As/kg-bw/day. Our results clearly demonstrate for this study population consuming around 500 g of cooked rice per day, that a cooked rice arsenic content above 200 μg/kg is - on its own - sufficient to give rise to significant amounts of genetic damage, even when there is little exposure through drinking water (Fig. 2).

### Genetic damage association with high arsenic rice is not confounded here by other factors

**Figure 3:**
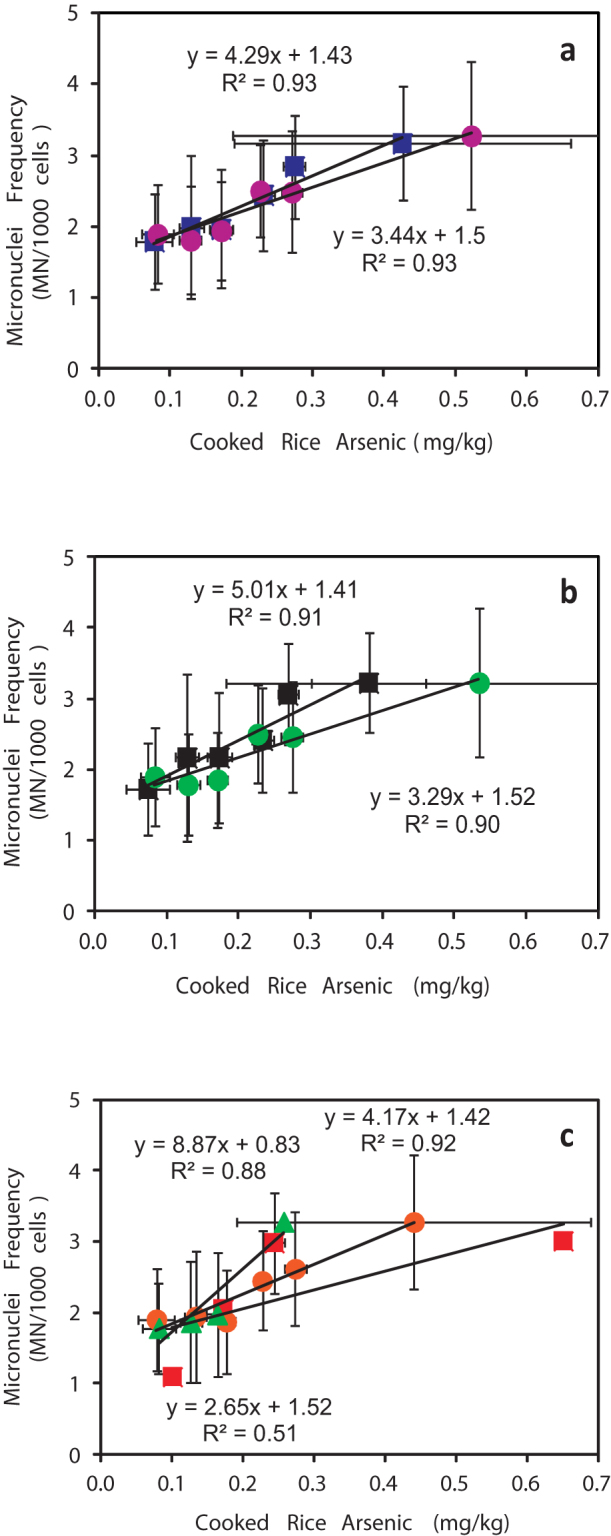
Cross-plot of urothelial micronuclei frequency (MN/1000 cells) and arsenic content in cooked rice for grouped data. (a) males (squares) and females (circles); (b) tobacco-users (squares) and tobacco-non-users (circles); (c) groups from each of the 3 study areas, viz. Murshidabad (squares), Nadia (circles) and East Midnapur (triangles). The linear best-fit trendlines are indicative only. Error bars represent ± 1 standard deviation for each parameter for each exposure group (A–F).

Age, gender, and tobacco-usage are often the major confounding factors in a genetic toxicity study, but the similar distribution of these factors throughout the groups suggests that it is unlikely for them to have substantially confounded the results. We note that the same positive relationship between micronuclei frequency and arsenic content of cooked rice is found in our study for both men and for women (Fig. 3a), for both tobacco-users and for tobacco-non-users (Fig. 3b), and for each of the 3 study areas (Fig. 3c). Questionnaire-based data shows that almost all of these individuals seldom travelled outside their local area and almost always used the same water source, thus suggesting that other sources of water are not a significant confounder (Table 1). Comparable body weight distributions in each study group act as a proxy variable that shows that the mean rice intakes of the different study groups were also comparable and, as such, the observed differences in genetic damage status in groups with >200 μg/kg in their consumed rice (Groups D, E, F) are, from a preliminary inspection, not likely due to differences in the level of rice intake (Table 1).

Following this preliminary inspection, in order to more robustly test whether or not key measured covariates, including age, gender, tobacco usage, bodyweight (as a proxy for cooked rice intake), drinking water arsenic, drinking water intake and study sub-area, are significant confounders to the association between MN and cooked rice arsenic, a stepwise (forwards/backwards) regression model was constructed utilising the data for the whole study population (i.e. without considering groups A–F). Distribution characteristics, including the scaling units, for these categorical and continuous covariates are listed in Table 2. The first step of this analysis (Table 3) revealed that the only covariates of significance in predicting MN, expressed as (MN/1000 cells)^1/2^, were, most significantly, cooked rice arsenic (p < 2.2e-16) and, to a much lower level of significance, tobacco usage (p = 0.022) with all of the following covariates tested not statistically significant (p > 0.05) confounders, viz. age (p = 0.648), body weight (p = 0.411), drinking water arsenic (p = 0.561), drinking water intake (p = 0.910), area (p = 0.126). The final model (Table 4) includes linear and quadratic cooked rice arsenic terms, tobacco usage and gender as covariates and indicates that gender is not a significant confounder (p = 0.704), tobacco usage is a weak confounder (p = 0.0478), whereas the overwhelming most important covariates in determining MN are the cooked rice arsenic linear (p = 4.50e-16) and quadratic (p = 1.43e-05) terms.

The elevated toxicity of inorganic arsenic species compared to organic arsenic moieties, such as arsenobetaine, arsenocholine, arsenolipids and arsenosugars, is well known^5^,^9^, but although we did not determine arsenic speciation in every cooked rice sample collected, the percentage inorganic arsenic (% i-As) content of a large sub-set of samples was relatively uniform and high (mean 88 ± 14%; SD; n = 92) in agreement with previous studies in the region^9^,^10^,^16^ and with only a weak (r^2^ = 0.24) correlation between % i-As and total arsenic, thus systematic variations in the percentage of inorganic arsenic in rice may also be eliminated as a significant confounding factor in this study.

Non-rice dietary sources of arsenic, for example from vegetables, fruit and seafood, may also be readily eliminated as significant confounders in this study. Drinking water intake of arsenic has already been shown to be low for the selected study group as a result of the study design. Halder et al.^17^ have shown that (i) the mean contribution to arsenic intake in rural West Bengal from vegetables is less than 0.4 μg/kg-bw/day, corresponding to less than 20% of total dietary exposure; and (ii) more importantly in relation to the present study, the inter-quartile range of such intake is less than 0.1 μg/kg-bw/day. Rowchowdhury *et al.*^18^ has previously shown that rice contributes over 90% of non-drinking water dietary exposure to arsenic in West Bengal.

Lastly, historical exposure is another potential confounding factor for other assays, but the employment of MN assay ensures that the results we obtained were only due to current exposure, since MN is not an inheritable property of a cell, rather each cell acquires MN during its short lifetime of being exposed to arsenic.

## Discussion

**Figure 4:**
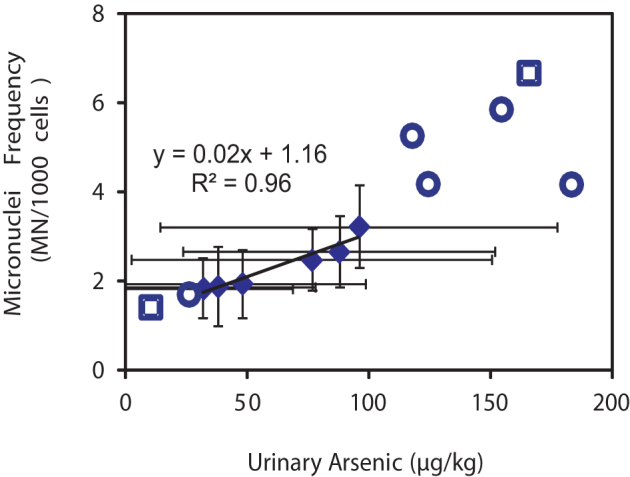
Cross-plot of urothelial micronuclei frequency (MN/1000 cells) and urinary arsenic content for grouped data. The linear best-fit trendline is indicative only. Error bars represent ± 1 standard deviation for each parameter for each exposure group (A–F). Data from this study (diamonds): for which arsenic exposure is predominantly from cooked rice and from Basu et al.^20^ (squares) and Ghosh et al.^19^ (circles), both for which arsenic exposure is predominantly from drinking water.

That the association observed here between micronuclei frequency in urothelial cells, and arsenic content in cooked rice is causal, is supported by (a) the strong positive correlation of mean urinary arsenic with mean cooked rice arsenic content amongst the groups (Fig. 1) (b) over the range of values observed, a strong linear correlation (r^2^ = 0.96) exists between mean urothelial micronuclei frequencies, and mean urinary arsenic content among the groups studied (Fig. 4); (c) the observed rate of increase in micronuclei frequency per unit increase in urinary arsenic is broadly similar to that previously observed for similar populations in West Bengal but exposed to arsenic largely through drinking water instead of rice^19^,^20^; (d) the previously inferred causal link between arsenic exposure from water and similar types of genetic damage^19^,^21^ and (e) the meta-analysis-based conclusion that micronuclei frequency is a meaningful predictor of cancer risk^22^,^23^. We therefore believe that, for populations, such as the one studied here, that may suffer from folate, animal protein, and vegetable fibre deficiency, all of which increase the risk of toxic effects arising out of chronic arsenic exposure^24^ and consuming rice as a staple, eating cooked rice with greater than 200 μg/kg of arsenic is unsafe. Although apprehensions regarding ill-effects of rice-derived arsenic exposure via the dietary route have been in existence for quite some time, never has this issue been addressed experimentally. Thus, ours is the first report which provides direct evidence that rice arsenic content on its own is associated with demonstrable genotoxic effects, and, in the case of the rural West Bengal population studied here, at arsenic concentrations greater than around 200 μg/kg. This study provides a basis for further similar but larger population based studies, using micronuclei frequency and/or other cytogenetic markers^22^,^23^ to elucidate the putative effects of other factors such as age (including with respect to *in utero*, and early childhood exposure)^25^,^26^,^27^, gender, genetic constitution^3^,^28^,^29^,^30^,^31^,^32^,^33^ and specific dietary elements^3^,^24^,^28^ on the relationship between arsenic exposure and toxicity – such studies would be particularly important to determine whether or not the genotoxic effects observed in our study are representative of impacts in populations, such as in the Americas, Europe, Africa and elsewhere in Asia, that are genetically different and often with better nutritional status.

For our study population, 200 μg/kg total arsenic was equivalent to approximately 180 μg/kg inorganic arsenic in rice and to a mean daily intake of inorganic arsenic of 2.0 μg/kg-bw/day. This intake value, above which we observe genotoxic effects, (i) is marginally lower than the PTWI (Provisional Tolerable Weekly Intake) of 2.1 μg/kg-bw/day previously recommended by the WHO; and (ii) lies in the region of the range of values (2.0–7.0 μg/kg-bw/day) reported by the Joint FAO/WHO Expert Committee on Food Additives (JECFA)^30^ as the inorganic arsenic BMDL (Benchmark Dose - Lower Confidence Limit) for a 0.5% increased incidence of lung cancer: our study is therefore strong vindication of EFSA's (European Food Safety Authority) concerns^29^ and JECFA's decision^30^ to withdraw the inorganic arsenic PTWI of 2.1 μg/kg-bw/day^30^. This study thus further highlights the inconsistency of current national and international regulation and guidelines for arsenic in drinking water, and rice^5^,^9^,^11^,^28^,^29^,^34^ as well as contributing to the increasing evidence^35^ that, irrespective of the exposure route, exposures to arsenic much lower than the equivalent of 2 L/day × 100 μg/L for a 65 kg person may result in significant genotoxic impacts. With over 3 billion people worldwide consuming rice as a staple^5^ and with over 10% of that rice in Bangladesh, Pakistan, and China; over 25% of that rice in Japan, Italy, France, Spain; and over 50% of that rice in USA and France estimated to have arsenic concentrations exceeding 200 μg/kg^5^, even taking into account variations in the proportion of inorganic arsenic in rice, the public health implications are considerable and warrant continued and further consideration of regulatory standards and other instruments^5^,^11^ to reduce public exposure to arsenic via this route.

In the areas investigated in this study, it is interesting to note that whilst about 40% (102 of 256) of the samples collected from highly groundwater-arsenic exposed areas showed cooked rice arsenic above 200 μg/kg, only about 2% (3 of 161) of the samples collected from relatively groundwater-arsenic unexposed areas showed the same elevated arsenic concentrations. These observations point to irrigation of rice paddy fields using high arsenic groundwaters as a significant cause of high As-accumulation in rice in West Bengal. Together with observed year-on-year seasonally-adjusted secular increases of arsenic in rice paddy soils in other similar parts of the Ganges-Brahmaputra-Megna basin^36^, this indicates that a further review of irrigation practices in these areas is warranted. Such a review would need to carefully address wider issues of how such practices might negatively impact crop yields and positively impact efficiencies of utilisation of water resources.

Despite the magnitude of the problem worldwide and the care required to ensure that changes in rice irrigation practices do not detrimentally impact on crop yields, it is worth noting that there are many effective and potentially effective management strategies for reducing arsenic exposure from rice. Suitable cooking methods^37^,^38^ and cooking with low arsenic waters can both somewhat reduce arsenic exposure. Management strategies to reduce arsenic accumulation in rice have been summarised by Meharg and Zhao^5^ amongst others and include promoting plaque formation and the use of aerobic cultivation processes^39^,^40^,^41^,^42^. Utilising the recently discovered route of rice uptake of neutral arsenous (III) acid through aquaporins that also serve as a channel for the uptake of silicic acid^43^,^44^, the use of suitable bioavailable silica fertilizer supplements might also be a productive strategy where costs allow^45^. Moreover, arsenic concentrations and the proportion of inorganic arsenic in rice varies widely, opening the opportunities to encourage the cultivation of relative low (inorganic) arsenic varieties^46^ or the genetic modification of rice^47^,^48^ to reduce arsenic accumulation in the grain^5^. So although >200 μg/kg rice in arsenic is associated with genotoxic effects in the studied cohort, which consumed rice as a staple, there do exist a variety of strategies whereby human exposure and subsequent health risks can be substantially reduced.

## Methods

### Study site, participants and sample collection

Study areas were selected in rural West Bengal because: (i) the dietary patterns are relatively homogeneous in rural West Bengal relative to the rest of India and particularly relative to much of the rest of the world^5^; (ii) rice is the staple for the local rural population; (iii) there is overwhelming uniformity in the methods used to cook rice, with over 90% of the population using a traditional method involving repeated washing and then boiling in excess water^49^ (iv) local rice was known to exhibit a wide range of arsenic contents^9^,^10^ and (v) arsenic in local rice is predominantly in the more toxic inorganic forms of arsenic^9^,^10^, thus minimising the requirement for arsenic speciation measurements. Partly in order to obtain a wide range of rice arsenic concentrations, we selected study areas in which rice paddy field irrigation waters had contrasting arsenic contents, viz. the very highly arsenic affected district of Murshidabad (Bhagwangola I block), the highly arsenic impacted district Nadia (Chakdha Block), and the relatively low groundwater arsenic district of East Midnapur (Khejuri I block). The selection criteria for the study participants have been described in detail previously^10^. For each of the study areas, the majority of samples were collected through informed medical camps organized by the CSIR-Indian Institute of Chemical Biology at pre-selected dates and times in which the villagers were requested to attend irrespective whether or not they exhibited any arsenic-attributable health symptoms such as arsenic-specific skin lesions. The balance of the samples were collected through randomised household surveys. Of those screened, criteria for inclusion in the study were: (i) using rice as a staple for at least the last 6 months; (ii) less than 10 μg/L arsenic in the household drinking water; (iii) spending over 80% of waking hours in the same area over the last 6 months; and (iv) agreeing to participate in the study by providing informed consent. Study participants consume the rice mainly which they grow in their own fields for at least for 6 months in a year. For the rest of the time, they consume local market-bought rice, which is grown in neighbouring fields, thus meaning that they consume locally grown rice throughout the year. Data regarding socio-economic factors, diet, tobacco usage and exposure history were collated by an experienced non-physician interviewer on the basis of a questionnaire. Each participant provided us with informed consent. From each study participant, drinking water, cooking water, and urine samples were collected for analysis (in this study, the drinking, and cooking water were the same for each participant and hence both referred to collectively henceforth as drinking water). Initially we screened the drinking water arsenic concentration of over 600 individuals, from which a group of 420 individuals met the screening criteria, including ≤10 μg/L of arsenic in their drinking water. Cooked rice samples were subsequently collected from these 420 individuals. Total arsenic and arsenic speciation in cooked rice was measured by inductively coupled plasma mass spectrometry ICP-MS and HPLC-ICP-MS after extraction following standardized protocols^10^. Based on the arsenic content in cooked rice and after the removal of data for 3 individuals for whom mis-coding errors were subsequently found, the remaining study participants (n = 417) were divided into 6 exposure groups with equally spaced classification boundaries as follows: Group A: ≤100 μg/kg; Group B: >100 μg/kg–≤150 μg/kg; Group C: >150 μg/kg–≤200 μg/kg; Group D: >200 μg/kg–≤250 μg/kg; Group E: >250 μg/kg–≤300 μg/kg; and, Group F: >300 μg/kg.

### Arsenic estimation

Analysis for total arsenic in water and rice was carried out at the Manchester Analytical Geochemistry Unit, University of Manchester by ICP-MS (Agilent 7500 Series ICP-MS). Total arsenic in lysed urine samples was analysed by ICP optical emission spectrometry (ICP-OES) (Perkin Elmer Optima 5300DV). Urinary arsenic concentrations were not corrected for creatinine content because creatinine has been shown to be marker for arsenic methylation efficiency and hence a possible confounding factor in our analysis^50^. Speciation of arsenic in rice was determined by ICP-MS coupled with HPLC. Preservation, preparation, rice digestion, and instrumental analysis methods were broadly as described previously^9^.

### Exposure assessment

Total daily intake of arsenic for each volunteer was calculated by: As_TDI_ = Σ As_i_ IR_i_; where As_TDI_ is the total daily intake; As_i_ is the measured arsenic concentration in the subscripted dietary item and IR_i_ the daily ingestion rate of that item; only cooked rice, cr, and water, w, were considered as important dietary items for arsenic intake for the study population^9^,^10^. IR_w_ was calculated from questionnaire data, specifically as the product of the volunteer's estimates of the number of drinks consumed each day and the volume of those drinks. Rice intake for each of men and women in rural populations in India have previously been shown to be a strong linear function of body weight^9^,^51^ and so IR_cr_ was calculated from measured body weight using previously published^9^ relationships: (IR_cr_/kg/day) = 0.01147 (BW/kg) (for males) or 0.010651(BW/kg) (for females) based on the diet survey data for West Bengal from the National Nutrition Monitoring Bureau (NNMB)^51^.

### Measurement of genetic damage by micronucleus assay

The effect of rice arsenic content on cellular damage was expected to be subtle, and hence, we have used the sensitive micronucleus assay (MN) in urothelial cells as a marker for chronic arsenic toxicity through rice. Micronucleus assay is an internationally well accepted cytogenetic method^19^,^20^,^21^,^22^,^23^,^29^,^52^,^53^,^54^,^55^ that has been extensively used for cancer risk assessment in humans^52^,^53^,^54^. Induction of genetic damage was quantified by employing MN assay in urothelial cells following a well standardized protocol^55^. Briefly, cells were isolated by centrifugation of urine, re-suspended in 0.9% NaCl and slides were prepared with 50 μl of cell suspension. The cells on the slide were fixed with methanol:acetic acid (3:1), stained with Giemsa and scored under the microscope following the criteria set down by Reali et al^55^. At least 1000 urothelial cells were scored per slide and 2 slides screened per individual.

### Statistical analyses

For preliminary assessment of the data, mean was used as the measure of central tendency and a one way ANOVA with a modified Tukey-Kramer Multiple Pairwise Comparisons Test, modified following the C-procedure of Dunnett^56^, adjusting for unequal group size and variance, was applied to test if the differences in the central tendencies of different parameters between the different study groups (A–F) were statistically significant or not after checking for within-group normality. Within-group normality was checked using a w/s test (α = 0.005) with the ratio of the group range to the group standard deviation used as the test statistic^57^, borderline (α ~ 0.005 to α ~ 0.05) normality tests were accepted for groups for which the sample size, n ≥ 30 (α ~ 0.005 was used for group D which showed less normality in the test for MN; and for groups B & E for urinary arsenic). Because of this non-normal pattern in one group of data, non-parametric tests were performed for checking and comparing possible changes in results and conclusions. For this, a Kruskal-Wallis test (α = 0.05) followed by, for each pair of groups, a Wilcoxon Rank Sum test (α = 0.05) with continuity correction was performed to make comparisons between the medians of the different study groups (A–F). To undertake a preliminary assessment of the impact of arsenic in cooked rice on micronuclei, we performed linear regression analysis with measured micronuclei frequency (/1000 cells) as the outcome and the arsenic in cooked rice (μg/kg), either as a categorical (groups A to F) or separately as a continuous variable as the predictor, with adjustment for gender, body weight, tobacco usage and drinking water arsenic at survey.

For linear regression modeling MN^1/2^ rather than MN was used as the outcome with the aim of complying with the normality assumption of the model. Shapiro-Wilks tests (α = 0.1) and Q-Q plots were used for testing normality in the data (and indicated that MN^1/2^ distributed more closely than MN to a normal distribution). The linear regression model using the categorical and continuous variables listed in Table 3 as the covariates was derived following a stepwise (forward-backward) model selection procedure. For selecting covariates, α_e_ = 0.10 was used as the criterion for entering variables into the model and α_o_ = 0.15 as the criterion for eliminating them; for checking linearity, for each continuous covariate, the significance of the coefficients of the linear, quadratic and cubic terms were tested in a stepwise procedure using the same α_e_ and α_o_ values as used in the previous step. Finally, a stepwise (forward-backward) selection procedure for all the paired interaction terms between covariates was followed using α_e_ = 0.05 and α_o_ = 0.10 - the reason for using a lower significance level than in previous steps was to ensure that interactions terms would be included, adding to the complexity of the model, only if they were strongly significant. The modified Tukey-Kramer analysis was carried out using the DTK^58^ package of R^59^, the KruskalWallis test was performed using the agricolae^60^ package of R, the stepwise linear regression analysis was carried out using the lm and step commands of R and GraphPad InStat software (GraphPad Software, San Diego). All other calculations were performed using Excel (Microsoft) (using appropriate data-type validation).

### Ethical approval

Ethical approval for elements of this study were obtained from the CSIR-Indian Institute of Chemical Biology and the University of Manchester Committee on the Ethics of Research on Human Beings.

## Figures and Tables

**Table 1:** Demographic characteristics and arsenic exposure status in the study population

	Group A	Group B	Group C	Group D	Group E	Group F	Total study population
**Cooked Rice Arsenic Range** (μg/kg)	≤100	>100 to ≤150	>150 to ≤200	>200 to ≤250	>250 to ≤300	>300	
**Number of samples**	113	118	84	35	30	37	417
**Gender**							
Male	49%	44%	50%	40%	50%	43%	47%
Female	51%	56%	50%	60%	50%	57%	53%
**Age** (years)	40.5	37.6	37.6	40.4	34.0	38.4	38.4
Mean (±SD)*	(±13.6)	(±12.5)	(±15.1)	(±12.0)	(±10.8)	(±13.6)	(±13.4)
**Tobacco Use**							
User	33%	26%	31%	29%	33%	35%	31%
Non-User	67%	74%	69%	71%	67%	65%	69%
**Body Weight** (kg)	49.1	49.8	52.4	51.3	54.5^a^	51.8	50.8
Mean (±SD)*	(±8.2)	(±7.7)	(±8.6)	(±6.3)	(±7.8)	(±9.6)	(±8.2)
**Time at Home** (Months/Year)	11.7	11.7	11.8	11.8	11.7	11.9	11.8
Mean (±SD)*	(±0.8)	(±0.9)	(±0.8)	(±0.6)	(±0.8)	(±0.2)	(±0.8)
**Arsenic exposure**							
Drinking water arsenic (μg/L)	3.5	3.4	4.2	5.7a,b	5.7a,b	5.0	4.1
Mean (±SD)*	(±2.8)	(±3.1)	(±3.2)	(±2.6)	(±3.2)	(±3.2)	(±3.1)
Drinking water intake (L/day)	3.0	3.3	3.1	2.9	3.8a,d,f	2.9	3.1
Mean (±SD)*	(±1.0)	(±1.1)	(±1.0)	(±0.8)	(±1.2)	(±0.8)	(±1.1)
Cooked rice arsenic (μg/kg)	80	129	170	226	273	480	174
(Mean ± SD)*	(±24)	(±16)	(±14)	(±14)	(±15)	(±295)	(±142)
Cooked rice intake (g/day)	540	550	580	560	600	570	560
Mean (±SD)*	(±90)	(±90)	(±100)	(±80)	(±80)	(±110)	(±110)
Urinary arsenic (μg/L)	32	38	48	76^a^	87a,b,c	96a,b,c	50
Mean (±SD)*	(±37)	(±40)	(±51)	(±74)	(±64)	(±81)	(±56)

*One-way ANOVA with modified Tukey-Kramer Multiple Comparisons Post Test.

^a^p < 0.05 compared to Group A.

^b^p < 0.05 compared to Group B.

^c^p < 0.05 compared to Group C.

^d^p < 0.05 compared to Group D.

^f^p < 0.05 compared to Group F.

**Table 2:** Whole group^a^ summary of categorical and continuous covariates used in linear regression model of micronuclei frequency (MN/1000 cells) in human volunteers living in the rural West Bengal study areas and consuming rice as a staple

Categorical covariates						
Covariate	Category 1	Number	Category 2	Number	Category 3	Number
Sex	Female	223	Male	194		
Tobacco User	Yes	127	No	290		
Area^b^	Very High	33	High	223	Low	161
Continuous covariates^c^						
Covariate	Unit	Min	Q1	Median (Mean)	Q3	Max
Cooked rice arsenic	μg/kg	5	100	147 (174)	200	1650
Drinking water arsenic	μg/L	0^d^	1	3 (4)	7	10
Drinking water intake	L/day	1.0	2.4	3.0 (3.1)	3.6	7.5
Age	year	15	27	38 (38)	48	85
Bodyweight	kg	33	45	50 (51)	55	77
Time at Home	months/year	6.0	12.0	12.0 (11.8)	12.0	12.0

^a^n = 417.

^b^Very High (groundwater arsenic hazard) = Murshidabad area; High (groundwater arsenic hazard) = Nadia area; Low (groundwater arsenic hazard) = Midnapore area.

^c^the following covariates were not included in the model: urinary arsenic (covariant with cooking rice arsenic), cooked rice intake (covariant with bodyweight).

^d^below 1 μg/L analytical method detection limit.

**Table 3:** Summary of first stage^a^ of stepwise selection of covariates to include in model of micronuclei frequency, (MN/1000 cells)^1/2^, for rural West Bengal study group consuming rice as a staple. None of the covariates – area, body weight, drinking water arsenic, age or drinking water intake are significant confounders for the association between MN and cooked rice arsenic

Covariate	Degrees of freedom	Sum of Squares	RSS	AIC	p (>χ^2^)	Sig. Code^b^
Model			256.52	−190.61		
Area	2	2.565	259.08	−190.46	0.126	
Body weight	1	0.414	256.11	−189.29	0.412	
Drinking water arsenic	1	0.208	256.31	−188.95	0.561	
Age	1	0.128	256.39	−188.82	0.648	
Drinking water input	1	0.008	256.51	−188.62	0.910	
Tobacco User	1	3.240	259.79	−187.38	0.0222	*
Cooked Rice Arsenic	1	56.291	312.81	−109.88	<2.2e-16	***

^a^(MN)^1/2^ ~ [Gender] + [Cooked Rice Arsenic] + [Tobacco User].

^b^Significance codes: 0 ‘***’ 0.001 ‘**’ 0.01 ‘*’ 0.05 ‘.’ 0.1 ‘ ‘ 1.

^c^Gender may be subsequently eliminated (see Table 4) because p(>χ^2^) > α_o_ = 0.15.

**Table 4:** Summary of model^a^ of micronuclei frequency, (MN/1000 cells)^1/2^, for rural West Bengal study group consuming rice as a staple

Covariate	Coefficient	Confidence Limits for Coefficient (2.5% & 97.5%)	Standard Error	t value	p (>|t|)	Sig. Code^b^
(Intercept)	1.151e+00	1.085e+00	3.336e-02	34.492	<2.2E-16	***
		1.216e+00				
Gender (Male)^c^	−1.113e-02	−6.873e-02	2.930e-02	−0.380	0.704	
		4.647e-02				
**Cooked Rice Arsenic**	**1.683e-03**	**1.292e-03**	**1.988e-04**	**8.464**	**4.59E-16**	***
		**2.074e-03**				
Tobacco-User	6.310e-02	6.641e-04	3.176e-02	1.987	0.048	*
		1.256e-01				
(Cooked Rice Arsenic)^2^	−6.990e-07	−1.012e-06	1.592e-07	−4.391	1.43E-05	***
		−3.861e-07				

^a^Residual standard error = 0.2666 on 412 degrees of freedom; Multiple-R^2^ = 0.2264; Adjusted R^2^ = 0.2189; F-statistic = 30.14 on 4 & 412 degrees of freedom, p-value < 2.2e-16; Shapiro-Wilk normality test for (MN)^1/2^ W = 0.9951; p = 0.2085.

^b^Significance codes: 0 ‘***’ 0.001 ‘**’ 0.01 ‘*’ 0.05 ‘.’ 0.1 ‘ ‘ 1.

^c^Gender listed for interest only; it is not (p = 0.7043) a significant predictor of MN^1/2^.
